# Robust Access Control for Secure IoT Outsourcing with Leakage Resilience

**DOI:** 10.3390/s25030625

**Published:** 2025-01-22

**Authors:** Khaled Riad

**Affiliations:** Computer Science Department, College of Computer Sciences & Information Technology, King Faisal University, Al-Ahsa 31982, Saudi Arabia; kriad@kfu.edu.sa

**Keywords:** Internet of Things (IoT), access control, leakage resilience, attribute-based encryption (ABE), outsourced decryption, master secret key protection, side-channel attacks, secure data outsourcing

## Abstract

The Internet of Things (IoT) has revolutionized various industries by enabling seamless connectivity and data exchange among devices. However, the security and privacy of outsourced IoT data remain critical challenges, especially given the resource constraints of IoT devices. This paper proposes a robust and leakage-resilient access control scheme based on Attribute-Based Encryption (ABE) with partial decryption outsourcing. The proposed scheme minimizes computational overhead on IoT devices by offloading intensive decryption tasks to the cloud, while ensuring resilience against master secret key leakage, side-channel attacks, and other common security threats. Comprehensive security analysis demonstrates the scheme’s robustness under standard cryptographic assumptions, and performance evaluations show significant improvements in decryption efficiency, scalability, and computational performance compared to existing solutions. The proposed scheme offers a scalable, efficient, and secure access control framework, making it highly suitable for real-world IoT deployments across domains such as smart healthcare, industrial IoT, and smart cities.

## 1. Introduction

The Internet of Things (IoT) is a network consisting of various intelligent devices and gadgets, including sensors, smartphones, and RFID tags [1]. The IoT has the potential to have a positive impact on our daily lives through applications such as smart city development, smart grid technologies, and advancements in healthcare services using intelligent medical devices [2]. In addition to providing convenience, the IoT has also stimulated its own advancement by enabling smart devices to monitor, collect, transmit, store, and share data within the IoT network. However, the increased connectivity and data sharing in IoT environments has raised significant security and privacy concerns [3]. IoT devices suffer from limited resources, which are represented in limited computational power and battery life. Consequently, traditional security mechanisms may not be directly applicable or efficient in IoT settings. Researchers have proposed various access control schemes for managing and securing IoT data [4,5]. Existing access control methods, such as lightweight encryption and cryptography algorithms, perform data evaluation on IoT devices, leading to security threats and efficiency issues.

As a result of the relevance and level of sensitivity of the IoT, information that is to be outsourced on untrusted cloud web servers requires effective access control in order to secure the information before it is outsourced [6]. In an open network environment, it is necessary to prevent unauthorized entities from accessing the information [7,8]. One of the primary access control methods for IoT is to leverage cryptographic techniques such as encryption and access control policies to protect the data. However, the limited computing and storage capabilities of IoT devices pose challenges in implementing robust and efficient access management protocols [9]. For IoT data, the sheer amount of data received from the IoT devices poses some challenges. Outsourcing data storage and processing to cloud or fog computing platforms has become an interesting solution to address the resource constraints of IoT devices [6]. However, the outsourcing of data and access control mechanisms introduces new security and privacy concerns, as data and access control policies are now managed by third-party service providers [10]. To address this issue, researchers have proposed the concept of secure outsourcing access control, where computationally intensive tasks are offloaded to more powerful cloud or fog computing entities, while IoT devices retain control over the data access [11].

When it comes to controlling data access, attribute encryption is a highly suitable choice. The innovation of attribute-based encryption determines whether the access should be granted or denied based on whether the users’ attributes fulfill the access control framework [12]. Consequently, there is now less reliance on cloud providers for handling data access control. Thus, this paper introduces a new form of secure IoT data outsourcing with resilient protection against leakage [13]. Attribute-Based Encryption (ABE) techniques have been extensively studied for secure data sharing in IoT. However, these schemes do not adequately address the issue of potential leakage of sensitive information when access control mechanisms are outsourced [14].

When initially considering cryptographic access control, the well-known Ciphertext Policy Attribute-Based Encryption (CP-ABE) method is often mentioned. However, ABE itself involves significant computational costs, particularly during decryption. To improve security performance in this phase, offline/online technology may be utilized [15]. Moreover, ABE schemes producing ciphertexts of constant size can help alleviate communication overhead, but they are not suitable for IoT terminals due to resource constraints [16]. Additionally, in an IoT environment where numerous terminals are deployed across distributed settings, several security issues such as side-channel attacks may arise when attackers gain access to the system’s master private keys [17]. If these keys are compromised, the entire system will lose its security integrity, and data privacy and safety within the system would face serious threats [18,19].

### 1.1. Motivation

The rapid proliferation of IoT devices has revolutionized multiple sectors, including healthcare, smart cities, and industrial automation. However, this growth has also introduced substantial challenges in managing the security and privacy of outsourced data, particularly when leveraging cloud and fog computing platforms for storage and processing. Traditional access control schemes, such as CP-ABE, offer fine-grained access control but suffer from computational overhead, scalability issues, and susceptibility to master key leakage. Furthermore, existing approaches often neglect the potential information leakage during outsourced computations, especially when sensitive access policies and cryptographic keys are managed by third-party servers. This oversight creates significant vulnerabilities in real-world IoT deployments, where resource-constrained devices cannot perform intensive cryptographic computations locally.

Recent advancements have focused on improving secure communication and data handling frameworks for distributed systems. For example, techniques explored in [20,21] provide innovative solutions for secure and efficient communication in dynamic environments. However, these works primarily address communication efficiency and sensor integration rather than fine-grained access control and resilience to key leakage. My proposed scheme builds on these foundations by introducing a leakage-resilient access control mechanism that offloads intensive computations to cloud servers while preserving the confidentiality of access policies and data. Unlike existing schemes, my approach integrates mechanisms for protecting against master secret key leakage and optimizes performance through partial decryption outsourcing. This ensures that even in resource-constrained environments, IoT devices can securely manage outsourced computations without sacrificing efficiency or scalability.

By addressing these critical gaps, my proposed access control model not only enhances the security of outsourced IoT data but also provides a scalable and computationally efficient framework that aligns with modern IoT security requirements.

### 1.2. Contributions

The main contributions in this paper are summarized as follows:

- This paper introduces a secure outsourcing access control model with leakage resilience to be used in the IoT environment, which takes advantage of attribute-based encryption in the access control.
- This paper designs efficient mechanisms to protect the access control scheme against potential information leakage when data and access policies are outsourced to third-party cloud or fog computing providers.
- This paper optimizes the computational and communication overheads of the access control scheme to accommodate the resource-constrained IoT devices by leveraging techniques such as offline/online encryption and decryption outsourcing.

Finally, comprehensive pilot evaluation of a variety of configurations confirmed the effectiveness and efficiency of the proposed access control system.

### 1.3. Acronyms and Notations

To facilitate understanding of the technical content, this paper provides a summary of the acronyms and notations used throughout this paper. Nomenclature and Abbreviations lists the key acronyms and notations, along with their respective descriptions. These definitions will help readers navigate the proposed scheme and its associated terminology with ease.

### 1.4. Organization

The remaining part of this paper is structured as follows: Section 3 introduces the system model and security requirements. Section 2 presents an overview of related studies and provides a comprehensive comparison between the CP-ABE schemes currently proposed and my access control solution. Section 4 outlines the fundamental preliminaries and notations that were taken into account during the development of my access control method. Section 5 explains the general description of my proposed model and the security model. In Section 6, this paper details the construction process for the proposed multidimensional access control scheme, including its five compatible algorithms. The security analysis and proofs are introduced in Section 7. An examination of the performance of the proposed scheme has been presented in Section 8. Finally, the paper summary is provided in Section 9.

## 2. Related Work

The problem of secure outsourcing access control with leakage resilience for the IoT has been studied in the literature [22,23,24]. Several existing works have proposed ABE schemes to achieve fine-grained access control in the IoT and cloud computing environments [24,25]. For example, ref. [22] presents an efficient decentralized attribute-based access control scheme for cloud storage, which supports user revocation. It incorporates a homomorphic encryption scheme to enable secure data storage and automatic data retrieval in the cloud [26]. However, these schemes do not consider the potential information leakage that may occur when the access control data and policies are outsourced to the cloud.

To address the information leakage issue, several research investigations have been introduced in this direction. Ref. [27] permits defining flexible access policies while efficiently revoking user attributes. However, the computational and communication overhead of these schemes may still be too high for resource-constrained IoT devices. More recently, ref. [28] proposed an efficient and secure attribute-based access control scheme for mobile cloud storage, which supports outsourcing of key generation and decryption to the cloud. This scheme reduces the computational overhead for IoT devices, but it does not consider the expected leakage of access control resources. Ref. [29] presented a leakage-resilient functional scheme. However, this scheme may not be suitable for IoT scenarios due to its high computational complexity.

The authors in [30] present a scheme called LR-ORCLS, which is relevant to the paper’s focus on secure outsourcing access control with leakage resilience. This scheme addresses the issue of leakage resilience in outsourced signatures, which is crucial for ensuring the integrity and authenticity of outsourced data. Ref. [31] contribute a detailed security analysis of for IoT environments. Their formal security verification using the AVISPA tool demonstrates the effectiveness of the proposed protocol in protecting against known attacks, aligning with the paper’s emphasis on secure access control mechanisms in the IoT. Ref. [32] categorize proposals related to access control in IoT into two main categories, focusing on ensuring the authenticity, confidentiality, and integrity of data streams during transmission. This categorization provides a broader perspective on access control mechanisms in the IoT, which is valuable for understanding the different approaches in the literature. Ref. [33] investigate leakage tracing enabled access control over outsourced data, emphasizing the importance of revoking leaked credentials and preparing judicial evidence. This aligns with the paper’s focus on leakage resilience and the need for robust access control mechanisms to address potential data breaches.

The authors in [34] propose a privacy-preserving smart IoT-based healthcare big data storage system with self-adaptive access control. This aligns with the paper’s focus on access control and security in IoT environments, particularly in sensitive domains such as healthcare, highlighting the importance of privacy-preserving access control mechanisms. Ref. [35] study the role of IoT security in managing enterprise human resource information leakage, emphasizing the need for the efficient and safe management of enterprise information. This reference provides insights into the broader implications of IoT security, including access control for sensitive enterprise data. Ref. [36] propose a scheme for a power IoT, which focuses on secure outsourcing decryption to resist selective ciphertext attacks. This contributes to the understanding of advanced encryption and access control mechanisms in IoT environments.

The authors in [37] present PROUD, a verifiable privacy-preserving outsourced attribute-based signcryption scheme supporting access policy update for cloud-assisted IoT applications. This scheme addresses the challenges of secure data designcryption process outsourcing, aligning with the paper’s focus on secure outsourcing access control. In summary, the related work section provides a comprehensive review of the literature on access control and security in IoT environments, encompassing various aspects such as leakage resilience, formal security analysis, privacy preservation, and encryption mechanisms. The synthesis of these references contributes to a deeper understanding of the current state of research in secure outsourcing access control for IoT. The work by [33] provides valuable insights into the security concerns related to outsourcing and cloud computing. It emphasizes the need for tracing leakage and access control over outsourced data, aligning with my focus on secure outsourcing access control with leakage resilience for IoT. The research serves as a foundational reference for understanding the importance of addressing potential data leakage and unauthorized access in outsourced environments, which is crucial for my study’s context.

The authors in [38] introduce a scheme, which addresses the challenge of ensuring confidentiality and access control for outsourced IoT data. This work is relevant, as it aligns with my focus on secure outsourcing access control for the IoT and provides insights into practical solutions for addressing confidentiality. The reference provided speaks to the task by offering a relevant and recent study that addresses IoT data outsourcing challenges. This work provides valuable insights and potential methodologies that can be compared and contrasted with the proposed scheme in the paper. The work by [39] presents a fine-grained access control mechanism for the Energy Internet, addressing the security challenges arising from data leakage in the collection, transmission, and storage processes.

The work by [40] is relevant to the current study. The paper discusses the delegation of computation tasks to untrusted cloud servers without compromising the confidentiality of the underlying data. This aligns with the current research’s objective of secure outsourcing access control with leakage resilience for the Internet of Things, as both works emphasize the importance of secure and scalable access control in cloud computing environments. The proposed methodology aligns with the work on access control for outsourcing risks [41]. This work discusses an access control model to mitigate the risks associated with outsourcing, which is relevant to the proposed methodology for securing access control in the context of outsourcing. The combination of cryptography and access control techniques in the proposed methodology resonates with the need for robust access control measures highlighted in this work.

The work by [42] addresses the security challenges introduced by edge computing in the context of e-health. The proposed modular exponential outsourcing algorithm and Mask Algorithm with blind pairs aim to preserve the privacy of sensitive data during outsourced computing. This reference is relevant to the task, as it discusses the use of outsourcing calculation verifiability, aligning with the theme of secure outsourcing access control for IoT devices. The proposed access control scheme in [24] is also related to my work. It addresses the need for secure data sharing and privacy protection in IoT by proposing a scheme that utilizes attribute authentication and threshold policy. Both papers emphasize the importance of access control, specifically focusing on the use of ciphertext attribute authentication and threshold policy to achieve secure data access.

The reference [43] discusses the use of CP-ABE for achieving fine-grained data access control in cloud-based IoT environments. This is relevant to the task, as it provides insights into the access control mechanisms for outsourced data, which is a key aspect of the paper’s focus on secure outsourcing access control for IoT. The use of attribute-hiding policy in CP-ABE aligns with the paper’s emphasis on leakage resilience, making it a valuable source for the Related Work section and detailed discussions. The reference provided by [44] discusses an efficient scheme. This is relevant to my work, as it addresses the issue of information leakage during data transmission, which is crucial in the context of secure outsourcing access control for IoT devices. By leveraging this approach, the proposed system can enhance the overall efficiency and security of data sharing, aligning with the objectives of the paper.

The work in [45] is relevant, as it discusses the secure outsourcing of Ciphertext Policy Attribute-Based Encryption, which aligns with my focus on secure outsourcing access control for the Internet of Things and provides insights into secure outsourcing mechanisms, which can inform my approach to ensuring leakage resilience in IoT access control systems. The work by [46] provides valuable insights into policy-based access control for constrained healthcare resources in the context of the IoT. The paper discusses the use of XACML in managing permissions within computer networks, which is highly relevant to the topic of secure outsourcing access control in IoT systems. Various other methods for carrying out gain access to administration in the cloud with security are produced collectively along two research lines: ABE and selective encryption processes, such as ([47,48]). Also, another set of contributions [49,50] applies access control in the cloud by introducing trust notation for granting or denying access.

As technological advancements in quantum computing pose significant threats to traditional cryptographic systems, it is crucial to assess whether the proposed access control scheme can withstand post-quantum adversaries [51]. Classical security primitives, such as AES-128, SHA-256, and ECDSA, rely on mathematical problems that can be efficiently solved by quantum algorithms, like Shor’s algorithm [52]. In contrast, post-quantum cryptographic primitives, including AES-256, SHA-384, and algorithms from the CRYSTALS suite, are specifically designed to resist quantum attacks [53]. The proposed scheme primarily relies on attribute-based encryption and bilinear maps, both of which are vulnerable to quantum adversaries. However, the modular structure of the proposed scheme allows for the underlying cryptographic primitives to be replaced or upgraded without requiring substantial changes to the access control logic. By substituting bilinear pairings and encryption components with post-quantum secure primitives, such as lattice-based encryption schemes, the proposed scheme can achieve quantum resilience while maintaining its functionality and efficiency.

In summary, existing access control schemes for IoT and cloud computing either do not consider the leakage resilience or do not provide efficient solutions for resource-constrained IoT devices.

## 3. System Model and Security Requirements

In this section, the detailed system model is presented, clearly defining the roles and responsibilities of each entity in the architecture to ensure clarity and coherence.

### 3.1. System Architecture

**Figure 1:**
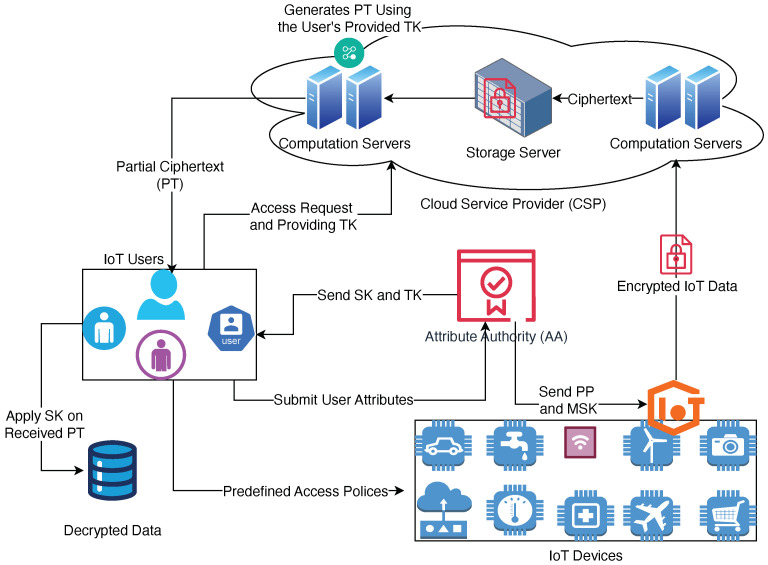
The system model showing the detailed communication steps among the four primary entities (Attribute Authority, Cloud Service Provider, IoT Devices, and IoT Users).

The proposed access control scheme operates within a cloud-assisted IoT system that consists of four primary entities, each with distinct responsibilities, as shown in Figure 1:

1. Attribute Authority (AA)
  - The AA is a trusted entity responsible for managing and distributing cryptographic keys and user attributes.
  - It generates the Master Secret Key (MSK), Public Parameter (PPs), user-specific Secret Keys (SKs), and Transformation Keys (TKs).
  - The AA ensures that the attributes assigned to each user align with their access privileges.
  - Security Role: Ensures attribute integrity and prevents unauthorized attribute assignment.
2. Cloud Service Provider (CSP)
  - The CSP provides storage and computational resources for encrypted IoT data.
  - It handles ciphertext storage and performs partial decryption operations on behalf of IoT devices using TK.
  - Security Role: Operates as an honest-but-curious entity, meaning it follows protocol specifications but may attempt to infer information from stored or processed data.
3. IoT Devices
  - IoT devices (e.g., sensors, actuators, and smart cameras) collect and generate sensitive data.
  - Due to limited computational power and energy constraints, IoT devices outsource intensive cryptographic computations to the CSP.
  - Security Role: Encrypt data before transmission, ensuring that sensitive information remains secure during storage and processing.
4. IoT Users
  - IoT users are authorized individuals or systems that need access to specific IoT data.
  - They possess user-specific SKs issued by the Attribute Authority.
  - Users download partially decrypted ciphertext from the CSP and perform final decryption locally to retrieve plaintext data.
  - Security Role: Ensure private key confidentiality and verify the correctness of decryption results.

### 3.2. Workflow Overview

The overall workflow of the proposed system can be summarized in the following key steps:

1. System Initialization:
  - The AA generates (PPs) and the (MSK), which are distributed appropriately.
2. Key Generation:
  - The AA generates user-specific SKs and TKs based on their attribute sets.
3. Data Encryption:
  - IoT devices encrypt collected data using PPs and predefined access policies.
  - Encrypted data (ciphertext) are then uploaded to the CSP for storage.
4. Partial Decryption by the CSP:
  - Upon receiving a decryption request, the CSP uses the TKs associated with the user to perform partial decryption and generates a Partial Ciphertext (PT).
5. Final Decryption by IoT User:
  - The IoT user downloads the PT and applies their SKs to retrieve the plaintext message.

### 3.3. Security Assumptions

- Trusted Attribute Authority: The AA is considered fully trusted and will not deviate from protocol specifications.
- Honest-but-Curious *CSP*: The CSP follows the protocol honestly but may attempt to infer sensitive information from stored ciphertexts and access policies.
- Secure Communication Channels: All communication between entities (e.g., IoT devices, CSP, AA, and IoT users) occurs over secure communication channels protected by standard encryption protocols (e.g., TLS).
- Device Integrity: IoT devices are assumed to be secure against unauthorized access, though side-channel attack resilience is implemented as an added layer of security.

### 3.4. Security Goals

The proposed access control scheme should satisfy the following security requirements:

- Fine-grained Access Control: The scheme should support flexible and fine-grained access control, where access policies can be defined based on multiple attributes of IoT users.
- Leakage Resilience: The scheme should be resilient against potential information leakage when the access control data and policies are outsourced to the cloud service provider.
- Efficiency: The scheme should be computationally efficient for resource-constrained IoT devices by offloading the heavyweight cryptographic operations to the cloud service provider.
- Verifiability: The IoT users should be able to verify the correctness of the decryption results returned by the cloud service provider.

### 3.5. Technical Challenges

To achieve the above security requirements, the proposed access control scheme needs to address the following technical challenges:

- Designing a fine-grained attribute-based access control mechanism that can be efficiently outsourced to the cloud.
- Incorporating leakage-resilient techniques to secure the access control data confidentiality and policies when they are outsourced.
- Reducing the computational and communication overheads of the access control scheme to accommodate the resource-constrained IoT devices.
- Ensuring the verifiability of the decryption results returned by the cloud service provider.

## 4. Scheme Preliminaries

In this section, the basic knowledge and assumptions are introduced on which this paper relies on to construct the proposed new access control:

- Composite order Bilinear Groups
  The first composite order bilinear groups were introduced by Dan et al. [54]. This scheme randomly selects groups of $N=p_{1}p_{2}p_{3}$ order with different prime numbers $p_{1}$, $p_{2}$, and $p_{3}$. Let *G* express the composite order group. Let $G_{1}$, $G_{2}$, and $G_{3}$ express the three subgroups with order $p_{1}$, $p_{2}$, and $p_{3}$, respectively. The bilinear map with computable non-degenerate $G\times G\to G_{T}$ is characterized by the following properties:
  – Non-degenetate: ∀g∈G$e\left(g,g\right)\neq 1_{G_{T}}$
  – Bilinear: $\forall x,y\in Z_{N}$ $e\left(g^{x},g^{y}\right)=e{\left(g,g\right)}^{xy}$
  To see this, if *g* generates *G*, $g^{p_{1}p_{2}}$ belongs to $G_{3}$, $g^{p_{1}p_{3}}$ belongs to $G_{2}$, and $g^{p_{2}p_{3}}$ belongs to $G_{1}$. $\forall \;a\in G_{1}$, and $\forall \;b\in G_{2}$, $e\left(a,b\right)=1_{G_{T}}$.
- Vector Notation
  Choose *G*. Then, $\vec{u_{1}},\vec{u_{2}}\in G^{n}$ is defined as follows:
  (1) $$e_{n}\left(\vec{u_{1}},\vec{u_{2}}\right)=\prod_{i=1}^{n}e\left(u_{1i},u_{2i}\right)\in G_{T}.$$
  where $\vec{u_{1}}=<u_{11},u_{12},\ldots ,u_{1n}>$ and $\vec{u_{2}}=<u_{21},u_{22},\ldots ,u_{2n}>$

**Definition** **1** (Discrete Logarithm Problem (DLP))**.** *Here is the finite field $Z_{p}$ generated by the generator g. Select a group number $h\in Z_{p}$, and find an group element $a\in Z_{p}$ such that $g^{a}=h$ whenever such integer exist.*

## 5. Model Definition and Security Model

### 5.1. Model Definition

The proposed scheme is the extension of the Lewko scheme [55]. In the scheme, the decryption stage needs a lot of computation. The proposed scheme outsources the calculation in the decryption phase:

- $Ini\left(1^{\lambda },U\right)\to \left(PP,MSK\right)$: This algorithm accepts λ (security parameter) as input and generates the PPs (public parameters) and the MSK (master secret key) as output.
- KeyGen(MSK,S)→(SK,TK): It accepts S∈U and the MSK as inputs. It then generates the $SK_{S}$ (secret key) of the user and the transformation key TK.
- Enc(PK,(A,ρ),M)→CT: This algorithm accepts the PK, the access structure (A,ρ) and the message *M* as inputs and then generates the ciphertext CT.
- Datadecryption: The decryption algorithm contains the partial decryption algorithm on the cloud server and the decryption algorithm on the client side.
  – $Dec_{out}\left(TK,CT\right)\to PT$: The algorithm accepts CT, which is associated with the (A,ρ) and the transformation key TK as inputs, and then outputs the partial ciphertext PT.
  – Dec(PT,SK)→m: The algorithm inputs the partial ciphertext PT and the user’s private key SK and outputs the message *M*.

### 5.2. Security Model

In this section, the scheme’s security has been defined through the game between *A* and *B*:

- Setup: The *B* process the $Ini\left(1^{\lambda },U\right)\to \left(PP,MSK\right)$ to obtain the PK and the MSK. Then, the PK will be sent to the adversary *A*. The challenger also initializes an empty set *D*, where $D\subseteq 2^{U}$, and an empty table *T*, where $T\subseteq I\times 2^{U}\times SK\times TK$ (*I* represents a handle counter and TK represents the transformation key).
- Phase 1: *A* can repeat the upcoming inquiries to *B*.
  – Create(S): *A* submits an attribute *S* to *B* to obtain the transformation key TK. *B* calls the KeyGen(MSK,S)→(SK,TK) to obtain the SK and the TK. *B* sets i:=i+1 and stores the entry (I,S,SK,TK) in *T*. Finally, TK is returned to the adversary.
  – Corrupt(I): When the *i*th exists in the table *T*, *B* can obtain the entry (I,S,SK,TK), and return SK to *A*. When no such entry exists, the algorithm will be terminated. Finally, *B* sets D:=D⋃S.
- Challenge: *A* sends a challenge access structure $A^{*}$, where the attribute set *S* in *T* is not satisfied with it, and two messages $M_{0},M_{1}$ with the equal length to the challenger. The challenger randomly selects the two messages $M_{0},M_{1}$ under $A^{*}$ to encrypt; it then sends $CT^{*}$ to *A*.
- Phase 2: It is the same phase 1 with the following restriction:
  – The attribute set whose private key will be asked is not satisfied with $A^{*}$.
  – *A* unable to request for decrypting of $M_{0}$ and $M_{1}$.
- Guess: The adversary outputs his guess $b^{\prime }$.

In this game, the advantage of the adversary is defined as

(2) $$Adv_{A}:=Pr\left|b^{\prime }=b\right|-\frac{1}{2}.$$

## 6. Scheme Construction

The concrete scheme with outsourcing decryption and anti-master secret key leakage is as follows:

### 6.1. System Setup

$Ini\left(1^{\lambda },U\right)\to \left(PP,MSK\right)$: This algorithm first selects a group of composite order $N=p_{1}p_{2}p_{3}$. Then, it randomly chooses the $\alpha ,a\in Z_{N}$ and $g_{1}$ which belong to $G_{1}$. Let *U* represent attributes. The algorithm selects random $s_{i}\leftarrow Z_{N},\forall i\in U$ and *n*, where $x_{1},x_{2},\ldots ,x_{n}\in Z_{N}$. The algorithm also chooses $t^{*},y_{1},y_{2},\ldots ,y_{n}\in Z_{N}$ and $\vec{\rho }\in Z_{N}^{n+1},\rho_{n+2}\in Z_{N},\forall i\in U\rho_{i}^{\prime }\in Z_{N}$ from the subgroup $G_{3}$.

Finally, The algorithm outputs PP: $PK=(N,g_{1},g_{3},g_{1}^{a},e{\left(g_{1},g_{1}\right)}^{\alpha },g_{1}^{x_{1}},g_{2}^{x_{2}},\ldots ,g_{1}^{x_{n}},\forall i\in U\;T_{i}=g_{1}^{s_{i}})$.

The master secret key MSK is as follows:

(3) $$\begin{array}{cc} MSK & =(U,\vec{K_{1}^{*}},L^{*},\forall i\in U\;K_{i}^{*})\\ \; & =(U,<g_{1}^{y_{1}},g_{1}^{y_{2}},\ldots ,g_{1}^{y_{n}},g_{1}^{\alpha }g_{1}^{at^{*}}\prod_{i=1}^{n}g_{1}^{-x_{i}y_{i}}>\cdot g_{3}^{\vec{\rho }},g_{1}^{t^{*}}g_{3}^{\rho_{n+2}},\forall i\in S\;T_{i}^{t^{*}}g_{3}^{\rho_{i}^{\prime }}). \end{array}$$

### 6.2. Key Generation

KeyGen(MSK,S)→(SK,TK): It accepts an attribute set S∈U and MSK. Then, it generates the SK and the TK. The algorithm randomly selects values $t,z,z_{1},z_{2},\ldots ,z_{n}\in Z_{N}$. The TK is then represented as follows:

(4) $$TK=(S,\vec{K_{1}^{z}},L^{z},\forall i\in S\;K_{i}^{z})$$

where

(5) $$\vec{K_{1}}=\vec{K_{1}^{*}}\cdot <g_{1}^{z_{1}},g_{1}^{z_{2}},\ldots ,g_{1}^{z_{n}},g_{1}^{at}\prod_{i=1}^{n}g_{1}^{-x_{i}z_{i}}>\cdot g_{3}^{\vec{\rho }},$$

(6) $$L=L^{*}g_{1}^{t}g_{3}^{\rho_{n+2}},$$

(7) $$K_{i}=K_{i}^{*}T_{i}^{t}g_{3}^{\rho^{\prime }}$$

The SK is represented as follows:

(8) $$SK_{s}=z^{-1}mod\;p$$

### 6.3. Data Encryption

Enc(PK,(A,ρ),M)→CT: This algorithm inputs the public key PK, an access structure (A,ρ), where *A* is a l×n matrix, and ρ is a mapping ρ(x)→U from rows of matrix to the attributes and a message *M*. It outputs the final ciphertext. It randomly chooses $\vec{v}=<s,v_{2},\ldots ,v_{n}>\in Z_{N}^{n}$ and generates $\lambda_{i}=v\cdot A_{i}$. It also selects $r_{x}\in Z_{N}$ for $A_{x}$. Then, it randomly chooses $r_{i}$ for i∈[1,…,l] and calculates $C_{i}$. The algorithm generates the partial CT.

(9) $$CT=(C_{0},\vec{C_{1}},{\left\{C_{i},D_{i}\right\}}_{i\in S})$$

as:

(10) $$C_{0}=M{\left(e{\left(g_{1},g_{1}\right)}^{\alpha }\right)}^{s},$$

(11) $$\vec{C_{1}}=<{\left(g_{1}^{x_{1}}\right)}^{s},{\left(g_{1}^{x_{2}}\right)}^{s},\ldots ,{\left(g_{1}^{x_{n}}\right)}^{s},g^{s}>,$$

(12) $$\forall i\;C_{i}=g^{a\lambda_{i}}T_{i}^{r_{i}},$$

(13) $$\forall i\;D_{i}=g_{1}^{r_{i}}$$

Finally, the CT is represented as

(14) $$CT=(C_{0},\vec{C_{1}},{\left\{C_{i},D_{i}\right\}}_{i\in S})$$

### 6.4. Data Decryption

The decryption algorithm contains the partial decryption algorithm on the cloud server and the decryption algorithm on the client side.

$Dec_{out}\left(TK,CT\right)\to PT$: The algorithm inputs the ciphertext CT associated with the access structure (A,ρ) and the transformation key TK and then outputs the partial ciphertext PT. It is a polynomial time to find a set $\sum_{i\in [1,\ldots ,l]}\omega_{i}\lambda_{i}=s$. This algorithm calculates the outsourced decryption ciphertext PT as

(15) $$PT=\frac{e_{n+1}\left(\vec{C_{1}},\vec{K_{1}^{z}}\right)}{\prod_{\rho (x)\in S}{\left(e\left(C_{x},L^{z}\right)e\left(D_{x},K_{x}^{z}\right)\right)}^{\omega_{i}}}=e{\left(g_{1},g_{1}\right)}^{\alpha sz}$$

The partial operations of the above equation are as follows:

(16) $$\begin{array}{cc} e_{n+1}\left(\vec{C_{1}},\vec{K_{1}^{z}}\right) & =e\left(\vec{C_{1}},\vec{K_{1}^{*z}}\right)\cdot e\left(\vec{C_{1}},\left(g_{1}^{z_{1}},g_{1}^{z_{2}},\ldots ,g_{1}^{z_{n}},g_{1}^{at}\prod_{i=1}^{n}g_{1}^{-x_{i}z_{i}}\right)\right)\\ \; & =e{\left(g_{1},g_{1}\right)}^{\alpha sz}e{\left(g_{1},g_{1}\right)}^{aszt^{*}}e{\left(g_{1},g_{1}\right)}^{aszt}\cdot e\left(C_{x},L^{z}\right)e\left(D_{x},K_{x}^{z}\right)\\ \; & =e{\left(C_{1},L^{*}\right)}^{z}e{\left(C_{1},g_{1}^{t}g_{3}^{\rho_{n+2}}\right)}^{z}e{\left(D_{x},k_{i}^{*}\right)}^{z}e{\left(D_{x},T_{i}g_{3}^{\rho^{\prime }}\right)}^{z}\\ \; & =e{\left(g_{1},g_{1}\right)}^{az\lambda_{i}t^{*}}e{\left(g_{1},g_{1}\right)}^{azt\lambda_{i}} \end{array}$$

If the attribute sets satisfy the access control policy, it will be able to decrypt correctly. Otherwise, it will not obtain the correct plaintext in the next step.

Dec(PT,SK)→m: The algorithm inputs the partial ciphertext PT and the user’s private key $SK_{S}$ and outputs the message *M*.

(17) $$m=\frac{C_{0}}{PT^{SK_{S}}}$$

## 7. Security Analysis

In this section, the in-depth security analysis of the proposed access control scheme is presented, focusing on its resilience against various potential attack vectors, including unbreakable DLP, side-channel attacks, master secret key leakage, and other known cryptographic vulnerabilities.

### 7.1. Unbreakable DLP

**Theorem** **1.** *Attackers cannot break the proposed system in polynomial time under the DLP.*

**Proof.**  The proposed scheme is an extension of the Lewko scheme [55], which is denoted by $\prod_{L}=\left(Setup_{L},Keygen_{L},Encrypt_{L},Decrypt_{L}\right)$. The scheme $\prod_{L}$ is $\left(l_{MSK},l_{SK}\right)$ master–leakage secure. The adversary cannot break it in polynomial time. If the adversary can break the DLP, the adversary can break the proposed scheme ∏=(Ini,Keygen,Encrypt,$Decrypt_{out},decrypt)$ in polynomial time. The stimulator *B* will interact with the challenger *C* of the Lewko scheme and the adversary *A*. □

Setup: It is easy to find that the system parameters of the proposed scheme in initialization stage are the same as those of the Lewko’s scheme. So, the stimulator *B* asks the challenger *C* of the scheme [55] about the system parameters. *C* returns the PK to *B*. Then, *B* sends it to *A*.

Phase 1: At this stage, when *A* sends *S* to B to obtain $SK_{S}$, *B* will send *S* to *C* and obtain the $SK_{S}$. The stimulator *B* selects a value $z\in Z_{N}$ randomly and computers $\vec{K_{1}^{z}},L^{z},\forall i\in S\;K_{i}^{z}$. Let $z^{-1}$ be the private key. The TK is represented as $TK=(S,\vec{K_{1}^{z}},L^{z},\forall i\in S\;K_{i}^{z})$. After that, it stores the entry (I,S,SK,TK) in table *T* and returns the transformation key TK and SK to the adversary *A*.

Challenge Phase: The adversary *A* sends to the *B* the messages $M_{0},M_{1}$ and the $A^{*}$. *B* selects b∈{0,1}. *B* sends $M_{b}$ and $A^{*}$ to the challenger *C*. The challenger *C* returns the ciphertext $CT=(C_{0},C_{1},{\left\{C_{i},D_{i}\right\}}_{i\in S})$. *B* sends CT to *A* as the challenge ciphertext $CT^{*}$.

Phase 2: This phase is the same as Phase 1 with restriction that the attribute set of which private key will be asked is not satisfied with $A^{*}$. The adversary is unable to request to decrypt $M_{0}$ and $M_{1}$

Guess: The adversary outputs his guess $b^{\prime }$.

If the adversary *A* has a probability advantage that cannot be ignored in this game, adversaries will solve the DLP. In other words, attackers cannot break the proposed system in polynomial time under the DLP.

### 7.2. Resilience Against Side-Channel Attacks

Side-channel attacks exploit physical information leakage during cryptographic operations, such as power consumption, electromagnetic emissions, or timing analysis, to deduce sensitive information like encryption keys or intermediate computation states. These attacks pose a significant threat to resource-constrained IoT devices, where operations are often conducted on limited hardware with fewer defenses against side-channel analysis. The proposed scheme incorporates several strategies to mitigate the risk of side-channel attacks:

- Outsourced Decryption: The most computationally expensive cryptographic operations, including bilinear pairing and modular exponentiation, are offloaded to cloud servers. This significantly reduces the number of resource-intensive computations performed on IoT devices, thereby minimizing the attack surface for side-channel adversaries.
- Constant-Time Algorithms: The cryptographic algorithms used in the proposed scheme have been implemented in a constant-time manner, ensuring that execution time does not vary with input values. This eliminates timing-based side-channel vulnerabilities.
- Transformation Key Isolation: The transformation key, used during the outsourced decryption phase, ensures that sensitive intermediate results are not exposed directly to the IoT device. Even if partial information is leaked from the IoT device, the master secret key remains secure, and the plaintext cannot be reconstructed without the transformation key and private key.
- Randomization of Intermediate States: During the key generation and decryption phases, random noise is added to the intermediate computation states, making it infeasible for adversaries to deduce meaningful patterns from observed emissions or power traces.

Proof of Security Against Side-Channel Attacks: Assume an adversary *A* attempts to extract secret information through side-channel analysis during decryption. Since the most vulnerable cryptographic operations are performed on the cloud server rather than the IoT device, the adversary’s view is limited to observing lightweight operations such as final partial decryption. Furthermore, randomized intermediate states and constant-time execution ensure that no meaningful side-channel information can be extracted during local computations. As such, the adversary cannot derive the master secret key or plaintext solely through side-channel information, even if they have physical access to the IoT device.

### 7.3. Resilience Against Master Secret Key Leakage

The proposed scheme integrates anti-leakage techniques by leveraging composite order groups and key splitting mechanisms. Even if partial information about the master secret key is exposed, the attacker cannot reconstruct the full key without access to additional secure components distributed across different domains. This multilayered key protection mechanism ensures the confidentiality and integrity of the access control system even under master key exposure.

### 7.4. Resistance to Collusion Attacks

Collusion attacks occur when multiple unauthorized users pool their attribute keys to gain illegitimate access to encrypted data. The proposed scheme prevents such attacks by assigning unique transformation and private keys to each user based on their specific attribute set. Even if multiple users collude and combine their attribute keys, they cannot bypass the access policy or reconstruct the plaintext without satisfying the defined access structure.

### 7.5. Resistance to Replay Attacks

Replay attacks involve an adversary that reuses previously intercepted messages to gain unauthorized access. In the proposed scheme, ciphertexts are tied to unique access policies and transformation keys, rendering intercepted ciphertexts useless in future sessions. The scheme also uses randomization during encryption to ensure that each ciphertext is unique, even for identical plaintext.

Finally, the proposed access control scheme demonstrates robust security properties against a wide range of attack vectors, including side-channel attacks, master secret key leakage, collusion attacks, and replay attacks. By offloading resource-intensive cryptographic operations to cloud servers, incorporating randomization techniques, and employing constant-time algorithms, the proposed scheme significantly reduces vulnerabilities in resource-constrained IoT devices. Furthermore, reliance on established cryptographic hardness assumptions ensures resilience against traditional and quantum cryptographic attacks. This comprehensive analysis confirms the effectiveness of the proposed scheme in maintaining data confidentiality, access control integrity, and operational security in IoT environments.

## 8. Performance Analysis

In this section, the comprehensive performance evaluation of the proposed access control scheme has been presented, comparing it with multiple existing state-of-the-art schemes, including the Lewko et al. [55] scheme, Chase et al. [56]’s multiauthority ABE scheme, and Zhang et al. [57] constant-size ciphertext ABE scheme. The evaluation focuses on key performance metrics, including encryption time, decryption time, ciphertext size, and computational overhead on IoT devices.

### 8.1. Evaluation Metrics

1. Encryption Time: Measures the time required to encrypt a plaintext message under an attribute-based access policy.
2. Decryption Time: Measures the time taken to decrypt ciphertext and retrieve plaintext, including both partial decryption (outsourced to CSP) and final decryption (on IoT users’ side).
3. Ciphertext Size: Represents the size of the encrypted data, directly impacting communication overhead.
4. Computational Overhead: Evaluates the computational burden on IoT devices, which is critical given their resource constraints.
5. Scalability: Assesses the performance impact when the number of attributes and users increases.

### 8.2. Comparative Analysis

The comparative results shown in Table 1 indicate the following:

- Decryption Time: The proposed scheme significantly reduced decryption time on IoT devices by outsourcing the most computationally intensive operations to the cloud server. In contrast, Lewko et al.’s scheme imposed a high computational burden on IoT devices during decryption.
- Ciphertext Size: Zhang et al.’s scheme achieved constant ciphertext size, which minimized communication overhead. However, it came at the cost of higher complexity in encryption. The proposed scheme maintained a good balance between compact ciphertexts and efficient encryption.
- Computational Overhead: Both the proposed scheme and Zhang et al.’s scheme exhibited low computational overhead on IoT devices, making them suitable for resource-constrained environments.
- Scalability: The proposed scheme is highly scalable due to its efficient attribute management and outsourced computation design, which ensures minimal performance degradation as the number of users and attributes increases.

### 8.3. Experimental Results

This paper conducted simulations to compare the proposed scheme against Lewko et al.’s scheme, Chase et al.’s scheme, and Zhang et al.’s scheme on an experimental setup emulating resource-constrained IoT devices (e.g., Raspberry Pi 4 with 4GB RAM and ARM Cortex-A72 CPU). The cloud environment was simulated using a high-performance server with multi-core architecture.

**Figure 2:**
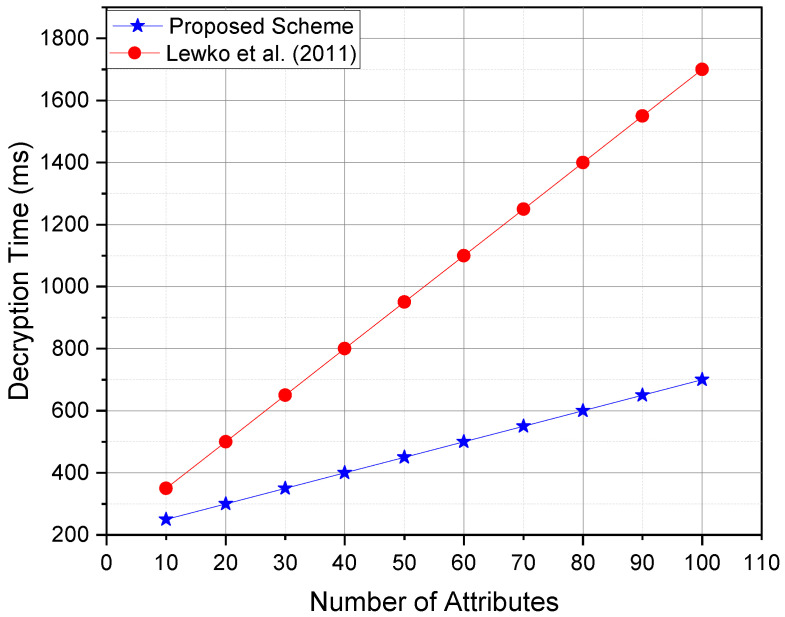
Decryption time against the number of attributes, ref. [55].

Figure 2—Decryption time vs. number of attributes:

- The proposed scheme demonstrated a significantly lower decryption time as the number of attributes increased thanks to the partial decryption outsourced to CSP.
- In Lewko et al.’s scheme, the decryption time scaled linearly with the number of attributes, causing performance degradation on the IoT devices.

**Figure 3:**
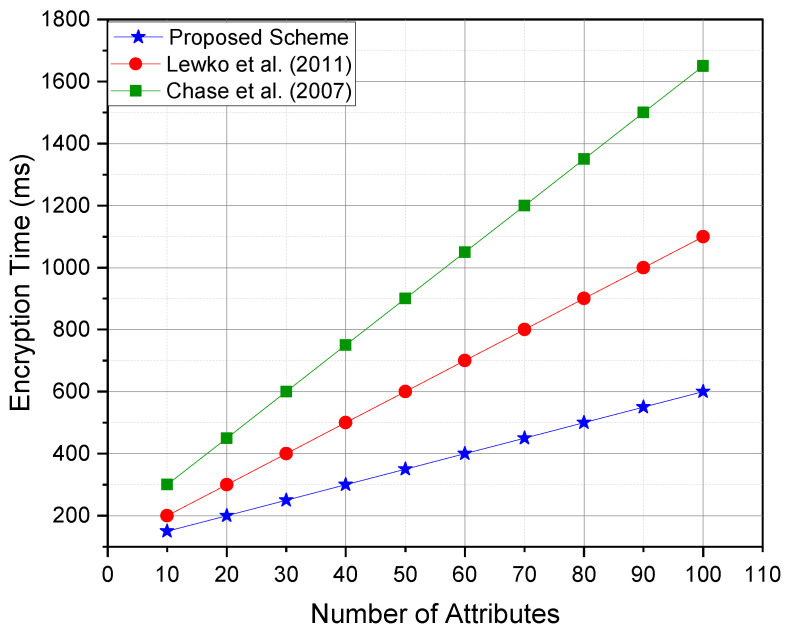
Encryption time comparison, refs. [55,56].

Figure 3—Encryption time comparison:

- The encryption time in the proposed scheme was moderate, and it remained competitive with Lewko et al.’s and Zhang et al.’s schemes.
- Chase et al.’s scheme exhibited higher encryption times, limiting its suitability for real-time IoT applications.

**Figure 4:**
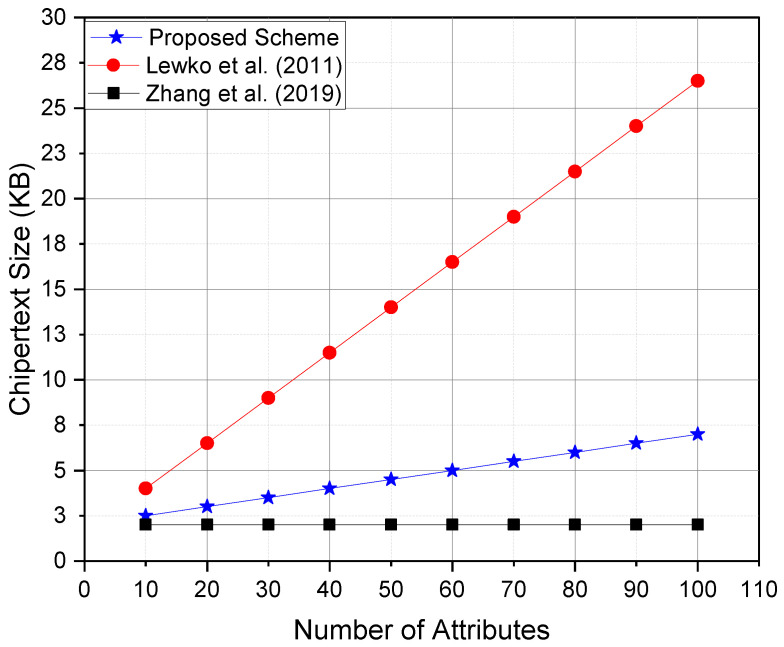
Ciphertext size comparison, refs. [55,57].

Figure 4—Ciphertext size comparison:

- Zhang et al.’s scheme achieved a constant ciphertext size, while the proposed scheme maintained compact ciphertexts with a slight increase as attributes grew.
- Lewko et al.’s ciphertext size grew significantly with more attributes, increasing storage and communication overhead.

Finally, the experimental evaluation and comparative analysis demonstrate that the proposed scheme achieves superior performance in terms of decryption efficiency, computational overhead, and scalability compared to existing access control schemes. By leveraging outsourced decryption and optimizing attribute management, the proposed scheme addresses the limitations of traditional attribute-based encryption models while meeting the stringent requirements of IoT environments.

## 9. Conclusions

This paper presents a leakage-resilient access control scheme designed for resource-constrained IoT environments. By leveraging ABE with partial decryption outsourcing, the scheme significantly reduces the computational burden on IoT devices while ensuring fine-grained access control and resilience against master key leakage and side-channel attacks. The introduced security analysis confirms the scheme’s robustness against common attack vectors, while performance evaluations demonstrate superior efficiency, scalability, and reduced computational overhead compared to existing solutions. The scheme is particularly well suited for real-world IoT applications, including smart healthcare, industrial IoT, and smart cities.

Future work will focus on integrating post-quantum cryptographic primitives, enhancing user privacy protections, and enabling cross-domain interoperability to further strengthen the scheme’s adaptability and resilience in evolving IoT ecosystems.

## Figures and Tables

**Table 1:** The detailed comparative analysis for the proposed scheme with three others schemes.

Scheme	Encryption Time	Decryption Time	Ciphertext Size	Computational Overhead (IoT)	Scalability
Proposed Scheme	Moderate	Low	Compact	Low	High
Lewko et al. [55]	Moderate	High	Large	High	Moderate
Chase et al. [56]	High	High	Moderate	High	Moderate
Zhang et al. [57]	Low	Moderate	Constant	Low	Moderate

## Data Availability

Data are contained within the article.

## Nomenclature and Abbreviations

Acronyms and notations:

**Symbol/Acronym**	**Description**
**Acronyms**	
ABE	Attribute-Based Encryption
AA	Attribute Authority
CSP	Cloud Service Provider
IoT	Internet of Things
DLP	Discrete Logarithm Problem
CP-ABE	Ciphertext Policy Attribute-Based Encryption
**Notations**	
λ	Security Parameter
*U*	Universal Attribute Set
MSK	Master Secret Key
PP	Public Parameters
SK	Secret Key
TK	Transformation Key
*A*	Access Structure (matrix)
ρ	Mapping from Attributes to Rows of *A*
CT	Ciphertext
PT	Partial Ciphertext
$g,g_{1},g_{3}$	Generators of Composite-Order Groups
e(·,·)	Bilinear Pairing
*s*	Random Secret Shared in the Sccess Structure
$v,\vec{v}$	Random vector used in Encryption
*z*	Random Value used in Key Generation
*M*	Plaintext Message
